# Study of Treatment with Intensified Omeprazole to Prevent High-Output Stoma—A Protocol for a Randomized, Parallel-Group, Open-Label, Superiority Trial in Adults Undergoing Ileostomy (STOP-HOS-1)

**DOI:** 10.3390/jcm15051841

**Published:** 2026-02-28

**Authors:** Tomasz Sylwestrzak, Michalina Ciosek, Katarzyna Połomska, Jarosław Kobiela, Piotr Spychalski

**Affiliations:** 1 Department of Gynecology and Obstetrics, Medical University of Gdańsk, M. Skłodowskiej-Curie 3a Street, 80-210 Gdańsk, Poland; 2 Department of Surgical Oncology, Transplant Surgery and General Surgery, Medical University of Gdańsk, M. Skłodowskiej-Curie 3a Street, 80-210 Gdańsk, Poland; michalina.ciosek@gumed.edu.pl (M.C.);

**Keywords:** omeprazole, PPI, ileostomy, stoma, high-output stoma, colorectal surgery

## Abstract

Introduction: High-output stoma (HOS) is a frequent and morbid complication following ileostomy formation. Proton pump inhibitors (PPIs) may reduce intestinal secretions, but no randomized trial has yet tested whether intensified intravenous omeprazole treatment prevents or mitigates early postoperative HOS. We aim to determine whether intensified PPI dosing reduces early postoperative ileostomy output compared with standard dosing. Methods and Analysis: STOP-HOS-1 is a randomized, parallel-group, open-label, superiority trial conducted at two academic centers in Poland. The target sample size is 100 adults undergoing the formation of a loop or end ileostomy. Participants will be randomized 1:1 to receive either: intensified omeprazole group—80 mg IV loading dose, followed by 40 mg IV twice daily through postoperative day (POD) 10, or standard omeprazole group—40 mg IV once daily through POD 10. The primary outcome is mean ileostomy output (mL/24 h) across POD 1–3. A ≥250 mL/day reduction is prespecified as clinically meaningful. Key secondary outcomes include: incidence of HOS (≥1000 mL/day for ≥3 consecutive days or ≥1400 mL on any single day), time to output stabilization (<1400 mL/day for 3 consecutive days), dehydration-related complications (hyponatremia, hypokalemia, acute kidney injury), length of hospital stay and 30-day readmission rate. The primary analysis will follow the intention-to-treat principle. One interim safety analysis is planned after enrollment of the first 20 patients. Discussion: Although PPIs are commonly used to reduce ileostomy output, high-quality evidence in the early postoperative setting is lacking. STOP-HOS-1 targets the critical period when output is most variable, and complications are most frequent, using a pragmatic randomized design and an objective, clinically meaningful primary endpoint. Conclusions: STOP-HOS-1 will provide the first randomized evidence on whether intensified postoperative PPI therapy reduces early ileostomy output and HOS-related morbidity, informing future standards of care.

## 1. Introduction

Formation of an end or diverting ileostomy is a commonly performed surgical procedure in abdominal surgery, especially in patients undergoing low anterior resection for rectal cancer, those with inflammatory bowel disease, and in surgical emergency settings such as small bowel obstruction, perforation, or small or large bowel ischemia, in which fecal diversion can mitigate sepsis risk and protect anastomoses [1,2].

In the United States, more than 100,000 new ostomies are created annually, with contemporary estimates approaching 150,000 per year [3]. In a large US Nationwide Inpatient Sample analysis of colorectal resections (2003–2014; n = 819,441), 16.6% required an ostomy, comprising 39.7% ileostomies and 60.3% colostomies [4]. Across the European Union, population-based registers report broadly similar distributions: in Sweden (2006–2019; n = 40,988), ileostomies accounted for ~40.0% of stomas (colostomy 47.9%, urostomy 12.0%) [5]. Stoma-related morbidity is common: across modern series, 21–70% of patients experience at least one complication [6]. For ileostomy in particular, early readmission is frequent; a meta-analysis reported an overall 30-day readmission rate of 20.9% and a dehydration-related readmission rate of 7.7% after ileostomy creation, consistent with US studies identifying dehydration and renal failure events as leading causes of early returns to the hospital [7,8].

One of the most troublesome early postoperative issues is the development of a high-output stoma (HOS), a complication for which definitions remain heterogeneous. Sources describe thresholds of stoma output from >1000 mL/day sustained for ≥3 days to >1500–2000 mL/24 h, with ~1400 mL/day frequently used as a pragmatic cut-off in the early postoperative period [9,10]. HOS occurs in approximately 20% of patients and is associated with dehydration, acute kidney injury and readmission [7,11]. The management of HOS includes dietary and behavioral interventions, antimotility agents (e.g., loperamide or codeine), fluid and electrolyte replacement, and identification and treatment of precipitating factors [9,12]. Guidelines emphasize restriction of hypotonic fluids, use of oral glucose–saline solutions, loperamide, and consideration of antisecretory therapy, including proton pump inhibitors (PPIs) within multimodal care pathways [13]. However, these strategies are commonly applied reactively rather than prophylactically and lack grounding in rigorous randomized clinical trials. There is a growing interest in proactive pharmacologic strategies that may modulate gastrointestinal secretions before HOS becomes established.

Pharmacodynamic data show that intravenous omeprazole produces rapid (1 h) and dose-dependent acid suppression, with a single-dose 40 mg iv sometimes insufficient to sustain pH > 4 during the first 24 h [14]. This supports a need for a loading dose and early iv twice-daily maintenance; this intensity is reflected in some HOS management guidelines, whereas other expert updates endorse PPIs without specifying dose, and no randomized trial has tested not only a twice-daily IV PPI regimen, but, in fact, any PPI regimen for HOS [12,15].

PPIs are routinely used perioperatively for gastric protection in surgical and critical-care populations [16,17]. Beyond gastric acid suppression, physiologic data suggest PPIs may reduce small-bowel secretions and alter luminal pH and transit, lowering effluent volume. Observational and physiologic studies in short-bowel syndrome and intestinal failure cohorts indicate that omeprazole reduces intestinal output and increases net water and sodium absorption, particularly in “net secretors” patients, in whom stomal effluent exceeds oral or enteral intake, typically more than 1.5 L per day in the early postoperative period [18,19]. To date, no randomized trial has evaluated intensified intravenous proton pump inhibitor therapy to prevent or mitigate high-output stoma after ileostomy formation.

The aim of this randomized, open-label, controlled trial (STOP-HOS-1) is to determine whether an intensified intravenous omeprazole regimen, compared with standard dosing, reduces early postoperative stoma output. The primary endpoint is the mean output across postoperative days 1 to 3. Secondary endpoints are the incidence of high-output stoma, dehydration-related complications, time to output stabilization, length of stay, and 30-day readmission rate. This manuscript follows SPIRIT 2025 guidelines for reporting clinical trial protocols [20].

## 2. Methods

Study design: This is a prospective, open-label, randomized, controlled, superiority trial conducted in two academic centers in Poland: The University Clinical Center in Gdańsk and the PCK Maritime Hospital in Gdynia. The trial is designed to evaluate whether an intensified regimen of intravenous omeprazole reduces postoperative ileostomy output in patients undergoing elective or emergency ileostomy formation compared with standard intravenous omeprazole treatment. The study adheres to the CONSORT 2025 guidelines [21].

Hypothesis: The trial tests whether an intensified intravenous (IV) omeprazole regimen (80 mg IV loading dose, then 40 mg IV twice daily for 10 days) is superior to standard dosing (40 mg IV once daily) in reducing early postoperative stoma output after ileostomy formation. The primary hypothesis is that intensified therapy reduces the mean 24 h ileostomy output across postoperative days (POD) 1–3.

Patient evaluation and selection: Participants are recruited at two academic centers in Poland (University Clinical Center, Gdańsk; PCK Maritime Hospital, Gdynia). Eligible adults undergo an elective or emergency loop or end ileostomy formation. Eligibility is confirmed by the attending surgeon using routine preoperative assessment (history, examination, standard laboratory tests and imaging as per local practice).

Inclusion criteria: Eligible participants are all adults (≥18 years) scheduled for the formation of a loop or end ileostomy—electively or emergently—who are able to provide written informed consent and have no contraindication to omeprazole or other proton pump inhibitors.

Exclusion criteria: Exclusion criteria comprise pregnancy or lactation; known hypersensitivity or allergy to omeprazole or to any other proton pump inhibitors; inability to provide written informed consent; concurrent participation in another interventional study likely to influence outcomes; pre-existing conditions that preclude accurate 24 h stoma output measurement; and perioperative circumstances expected to prevent reliable monitoring, including planned or immediate postoperative admission to the intensive care unit (ICU) with an anticipated ICU stay of more than 48 h, or any other critical illness in which early stomal output is frequently absent. In addition, any situation judged by the investigator to pose unacceptable risk or to confound the assessment of the primary endpoint will result in exclusion. Inclusion and exclusion criteria are presented in Table 1.

Randomization and allocation: Participants are randomized 1:1 (intensified vs. standard dosing) with stratification by admission type (elective vs. emergency), using computer-generated permuted blocks of variable sizes (4, 6, or 8) within each stratum to maintain temporal balance while minimizing allocation predictability. Additional stratification by small bowel resection length was considered but not implemented due to the expected low number of patients with resections ≥50 cm, which would risk creating sparsely populated strata and imbalance in smaller subgroups. Allocation of concealment is ensured via sequentially numbered, opaque, tamper-evident sealed envelopes prepared centrally by a study coordinator who is independent of recruitment, enrolment, and clinical care. Envelopes are identical in appearance, made of non-transparent material, and stored in a secure, access-restricted location within the ward. Each envelope is opened in strict numerical order only after confirming patient eligibility and completing baseline data collection. The envelope method was chosen to enable real-time randomization in a surgical setting while maintaining adequate allocation of concealment. Randomization and allocation are performed by the on-call surgeon or, alternatively, by a study investigator, following training in eligibility assessment, enrollment timing, and the envelope procedure. Due to the nature of the intervention—intravenous omeprazole dosing with specific timing and preparation requirements—and the impracticality of preparing indistinguishable placebo infusions in a busy surgical ward or emergency setting, the blinding of participants and treating clinicians was not feasible. The study is therefore designed as an open-label trial.

To minimize performance and measurement bias, the primary endpoint, mean daily stoma output during postoperative days 1–3, is an objective, quantitative, and routinely charted parameter measured by nursing staff as part of standard postoperative care, independent of the study team. Measurements are entered directly into the electronic medical record according to established hospital protocols and verified daily by the attending surgeon. Nursing staff responsible for output measurement are not involved in treatment allocation or study hypotheses, reducing the likelihood of differential reporting. Finally, data extraction and statistical analysis are conducted by investigators blinded to group allocation, in accordance with a pre-specified statistical analysis plan, further mitigating potential bias.

Data collection and management: Data on demographics, surgical details, laboratory values, stoma output, complications, and readmissions will be recorded using standardized electronic case report forms (eCRFs). All data will be pseudonymized and stored on secure, password-protected external hard drives. Quality control will be maintained through periodic source data verification.

Interventions:

Participants randomized to the intensified omeprazole arm receive an 80 mg intravenous loading dose on the day of surgery, followed by 40 mg intravenously twice daily (06:00 and 18:00) to complete a 10-day course. The loading dose is used to maximize early pharmacodynamic separation within the POD 1–3 primary endpoint window, as intravenous omeprazole exhibits dose-dependent antisecretory effects, with controlled pH studies demonstrating faster and more reliable achievement of intragastric pH ≥ 4 with 80 mg compared with 40 mg [22,23]. If discharged before POD 10, treatment continues orally at the same dosing frequency until POD 10.

Participants in the control arm receive omeprazole 40 mg intravenously once daily (06:00) during hospitalization only, without post-discharge continuation, in accordance with standard ward prophylaxis protocols. Administration times may be adjusted by ±1 h for operational reasons. All administered doses and any deviations (including timing, route of administration (intravenous vs. oral after discharge), dose modifications, missed doses, and premature discontinuation) from the planned regimen are prospectively recorded in the eCRFs. Details of the intervention are presented in Table 2.

Outcome assessment: Ileostomy output is measured in routine postoperative care by trained nursing staff using standard ward measuring containers and routine bedside measurement procedures. Measurements are recorded in the electronic case report form at predefined intervals. Patients are not overtly labeled by treatment allocation, and nursing staff are not actively informed of group assignment at the time of outcome recording. Although staff may be able to infer allocation from clinical documentation, outcome measurement relies on objective volume measurements, and measurement precision is expected to be comparable across study arms. Formal inter-observer reliability testing is not implemented within the trial framework.

Concomitant care (both arms): Postoperative supportive care in both treatment arms is delivered within a standardized monitoring and decision framework implemented at the participating centers. All patients undergo uniform 24 h intake–output charting and predefined laboratory surveillance, including daily assessment of serum sodium, potassium, magnesium, and renal function during the early postoperative period. Clinical staff follow identical monitoring protocols and predefined escalation triggers, minimizing the risk that differences in clinician behavior influence outcomes, rather than the intervention itself. As electrolyte disturbances, including hypomagnesemia, are recognized as adverse effects of prolonged proton pump inhibitor therapy [24,25], daily postoperative laboratory monitoring provides safety oversight during the temporary 10-day dose intensification, enabling early detection and management of clinically relevant abnormalities, all of which are recorded in the eCRF [26].

Postoperative care follows standardized ward pathways for fluid and electrolyte management after ileostomy formation. Patients receive structured dietary counseling, including restriction of hypotonic fluids and preferential use of oral glucose–saline rehydration based on the World Health Organization formulation [27].

The standard WHO solution composition includes: 75 mmol/L sodium, 75 mmol/L glucose, 20 mmol/L potassium, 65 mmol/L chloride, and 10 mmol/L citrate, resulting in a total osmolarity of approximately 245 mOsm/L.

Where clinically indicated, temporary nil per os management, restrictive intravenous fluid therapy with stepwise reintroduction of oral intake, or initiation of total parenteral nutrition may be applied according to institutional standards. Routine thromboprophylaxis, analgesia, and antimicrobial prophylaxis are administered as clinically indicated.

Within this standardized framework, supportive interventions—including fluid therapy, electrolyte supplementation, dietary measures, and pharmacological treatment—are not protocolized in fixed doses, allowing for adjustment to the severity and dynamic course of high-output stoma and individual clinical status [11,28]. The use and dosing of antimotility agents (e.g., loperamide, with adjunctive codeine when clinically indicated) cannot be governed by a rigid algorithm in the early postoperative period, as the escalation of therapy must be individualized according to stoma output dynamics and overall clinical condition. Consequently, antimotility treatment is guided by routine clinical judgment rather than fixed study-mandated dosing schedules. Randomization is expected to partially balance baseline susceptibility to high-output stoma between groups.

All co-interventions, including antimotility therapy, as well as any deviations from the standardized pathway, are prospectively recorded in the electronic case report form. This approach enables transparent post hoc assessment of co-intervention patterns and potential between-group imbalances and allows for sensitivity analyses to evaluate the robustness of the estimated treatment effect to differences in supportive pharmacotherapy while preserving real-world external validity.

Management of intervention discontinuation: In the event of adverse events requiring discontinuation of the intervention, such as suspected hypersensitivity or allergic reaction, omeprazole administration will be stopped immediately, and the patient will receive appropriate supportive care in accordance with local clinical protocols. As both study arms receive the same active pharmaceutical ingredient (omeprazole), differing only in dosage and duration, participants are, therefore, not exposed to additional pharmacological risks attributable to group allocation. No crossover or alternative treatment will be administered following discontinuation.

Surgical approach: The decision to create an ileostomy and the operative technique (loop vs. end; laparoscopic vs. open), including conversion to an open approach, follows standard clinical practice and remains at the discretion of the performing surgeon; intraoperative deviations and complications are documented. The trial includes both loop and end ileostomies; although baseline output may differ between types, randomization is expected to balance their distribution between study arms, preventing systematic confounding of the treatment effect. As the primary analysis is based on between-group comparison, variation in absolute output levels does not bias treatment estimates unless ileostomy type acts as an effect modifier, for which no evidence currently exists.

To account for anatomical determinants of stoma output and identify potential short-bowel outliers, the operating team records ileostomy type, surgical approach, length of small bowel resected, estimated residual small-bowel length, and the distance of the stoma from the ligament of Treitz. These variables are prospectively captured in the eCRF and will be used for prespecified exploratory subgroup and treatment-by-covariate interaction analyses; any observed effect modification will be considered hypothesis-generating.

Postoperative management and follow-up: Stoma output (mL/24 h) is measured daily by ward staff from POD 1 to POD 10 (or until discharge), with secretion-management measures applied as previously described. Output measurements recorded during ICU stay are included when available. In the event of death occurring after ileostomy formation within POD 1–3, available output data up to the time of death are included, and no post-mortem values are imputed. After discharge, output is recorded by the patient (or caregiver) following standardized training delivered by ward personnel, which is provided to all new ileostomy patients as part of usual care, irrespective of trial participation. Post-discharge monitoring includes scheduled ambulatory follow-up (with laboratory testing, including sodium and potassium, obtained when clinically indicated) and structured telephone contacts on POD 14 and POD 30 conducted by investigators who do not provide medical advice (to avoid influencing readmission rate or adverse-event reporting); calls are used solely to ascertain adverse events, readmissions, and reported stoma output volume.

Criteria for discharge: Discharge requires that the patient can eat independently, is mobilized to a level appropriate for the preadmission baseline, and has stable, controlled stoma effluent with no features of HOS. Inflammatory markers must be stable without an upward trend. The patient must be independent of TPN, unless discharge is to hospice care or the patient has been enrolled in a home parenteral nutrition program. The final decision to discharge rests with the responsible attending physician. Patients’ flowcharts are presented in Table 3 and Table 4.

## 3. Analysis

Endpoints:

The primary endpoint is obtaining a clinically significant reduction in mean daily ileostomy output (mL/24 h), defined as a reduction of ≥250 mL/day, across POD 1–3. Key secondary endpoints include the incidence of HOS, defined as stoma output of ≥1000 mL/day for 3 or more consecutive days or ≥1400 mL/day on any single day during the index admission; time to output stabilization (<1400 mL/day for 3 consecutive days); dehydration-related complications, specifically hypokalemia (serum potassium < 3.5 mmol/L), hyponatremia (serum sodium < 135 mmol/L), acute kidney injury (AKI, defined using KDIGO 2012 criteria as a rise in serum creatinine by ≥0.3 mg/dL within 48 h, or ≥1.5 times baseline within 7 days, or urine output < 0.5 mL/kg/h for 6 h) [29]; length of stay for the index admission; 30-day readmissions; and total days hospitalized within 30 days. Exploratory analysis will describe outcomes by stratum (elective vs. emergency patients). To reduce performance and measurement bias, the primary endpoint—mean daily stoma output during POD 1–3—was selected as an objective parameter routinely recorded by nursing staff as part of standard postoperative care, independent of the study team.

This interval captures the phase of greatest physiological variability and highest risk of fluid and electrolyte disturbances while minimizing missing data and heterogeneity introduced by early discharge, as many patients are discharged within the first postoperative day. Restricting the primary endpoint to POD 1–3, therefore, maximizes data completeness and internal validity while avoiding any influence of outcome ascertainment on discharge decisions. Measurements obtained during POD 1–10 will be analyzed as secondary and exploratory outcomes, with longer-term clinical outcomes assessed through POD 30 follow-up.

Output volumes are documented in the electronic medical record according to established institutional procedures. Nursing staff are not involved in treatment allocation or study hypotheses, and data extraction and statistical analyses are performed by investigators blinded to group assignment under a prespecified statistical analysis plan.

Sample size calculation:

The sample size was determined using institutional retrospective data supported by published clinical literature. A preliminary analysis of 20 patients undergoing ileostomy formation in 2024 at the University Clinical Center in Gdańsk demonstrated a mean POD 1–3 output of 1160 mL/day (SD = 360 mL), with 17 complete datasets available. To account for uncertainty inherent to small preliminary samples, the standard deviation was conservatively inflated to 400 mL for planning purposes. Under these assumptions and using a two-sided t-test (α = 0.05, power = 80%), the estimated sample size was 41 participants per arm. After accounting for an anticipated 15% dropout rate, the required enrollment increased to 49 participants per arm (n = 98 total). To minimize the risk of underpowering due to variance misestimation, target enrollment was increased to 50 participants per arm (n = 100 total). Sensitivity analyses demonstrated preservation of 80% power for SD values up to 415 mL. With 50 participants per arm, statistical power remains 87.5% assuming SD = 400 mL and ≥83% for SD ≤ 425 mL, indicating robustness to moderate deviations from planning assumptions. The assumed 15% attrition rate is supported by institutional experience from comparable perioperative trials. Analyses will follow a modified intention-to-treat principle, with all exclusions transparently documented to limit attrition bias. Variance assumptions will be reassessed at a preplanned interim analysis focused solely on variability estimation; if observed dispersion materially exceeds projections, mitigation strategies such as extended recruitment will be considered without evaluation of treatment effect, thereby preserving statistical validity.

No randomized trial has established a validated minimal clinically important difference for postoperative ileostomy output. Therefore, the calculation was anchored to clinically meaningful output thresholds used in routine practice to define high-output stoma, commonly defined as output exceeding 1.4 L/24 h and associated with clinically significant fluid and electrolyte disturbances requiring medical intervention [30]. As stoma output represents an objective and routinely recorded parameter, the primary endpoint is considered minimally susceptible to assessment bias despite the open-label design.

In the institutional cohort, a reduction of 250 mL/day corresponds to 20–25% of typical POD 1–3 output volumes and is sufficient to shift a substantial proportion of patients below commonly applied high-output thresholds, which trigger intensified monitoring and therapeutic escalation in routine care. The selected effect size, therefore, reflects a clinically actionable and patient-relevant improvement rather than a purely statistical difference.

Interim analysis:

An interim analysis will be performed after 20 patients total (10 per arm) have completed postoperative days 1–3 assessment and have primary outcome data available. This interim timepoint corresponds to approximately 25% of the target enrollment. This analysis will assess feasibility, safety, and conditional power. A Haybittle–Peto boundary will be applied for early stopping due to efficacy (*p* < 0.001, two-sided) [31], while recruitment may be discontinued for futility if conditional power to detect the prespecified 250 mL/day difference falls below 20%. As futility stopping may increase the risk of type II error, any decision to discontinue recruitment will be interpreted cautiously and will considerconsistency of observed trends together with clinical plausibility rather than conditional power alone.

The interim review is primarily safety-focused and will be conducted by an independent statistician not involved in trial conduct. Given the modest sample size and the well-established perioperative safety profile of proton pump inhibitors, rigid numerical stopping thresholds for infrequent adverse events are not prespecified. Instead, the review will include descriptive assessment of serious adverse events, clinically relevant electrolyte disturbances (including hypomagnesemia, hyponatremia, and hypokalemia), acute kidney injury, and any unexpected safety signals potentially related to intensified omeprazole therapy. Any clinically meaningful imbalance in serious or treatment-related adverse events may prompt trial modification, temporary suspension, or early termination in accordance with Good Clinical Practice and institutional safety oversight procedures. Interim results will remain confidential and will not be disseminated as a standalone report.

Statistical analyses:

The primary analysis will follow a modified intention-to-treat principle, defined as all randomized participants who underwent ileostomy formation as planned and had stoma output recorded for at least two of the first three postoperative days. The primary endpoint (mean POD 1–3 stoma output) will be calculated using all available daily measurements within this window, with limited missingness handled as specified below.

Continuous variables will be summarized as means with standard deviations or medians with standard deviations or medians with interquartile ranges per distribution; categorical variables as frequencies and percentages. All estimates are presented as two-sided 95% confidence intervals.

The primary efficacy model was prespecified as a parsimonious model including a limited number of covariates in order to preserve statistical power and reduce the risk of overfitting, given the planned sample size. Randomization is expected to achieve a balance of baseline anatomical and clinical characteristics between study groups. Variables potentially influencing absolute stoma output, including resected small-bowel length, study center, use and dose of antimotility agents, and oral or enteral intake volumes, will be prospectively captured in the eCRF. Because the selected post-randomization variables (particularly antimotility use and intake volume) may lie on the causal pathway of the intervention, they will not be routinely included as adjustment covariates in the primary model to avoid the introduction of bias through overadjustment. Instead, these factors will be examined in prespecified exploratory and sensitivity analyses to assess the robustness of findings and potential heterogeneity of treatment effect.

Secondary outcomes will be analyzed as follows: the incidence of high-output stoma will be compared using the chi-square test; time to output stabilization will be assessed with Kaplan–Meier estimates, the log-rank test, and the Cox proportional hazards model; the incidence of hyponatremia and hypokalemia will be compared using the chi-square test; total potassium supplementation and both index and total length of hospital stay will be compared using two-sided *t*-tests. Prespecified exploratory subgroup analyses will be conducted according to surgical urgency (elective vs. emergency), ileostomy type, and selected baseline characteristics. As the trial is not powered to detect interaction effects, subgroup analyses will be considered hypothesis-generating and interpreted cautiously.

Handling of missing data:

Missing daily stoma output measurements within the POD 1–3 primary endpoint window may occur due to early discharge, ICU transfer, or clinical deterioration.

Cases with two or more missing daily measurements within the primary endpoint window will not be included in the primary analysis and will be reported transparently. In the event of death within POD 1–3, only stoma output data collected up to the time of death will be included.

Missing data will be imputed using multiple imputation by chained equations (MICE), generating 50 datasets under the missing-at-random (MAR) assumption.

The model will include the treatment arm, stratification variable, baseline covariates, observed outputs, co-interventions, and laboratory parameters. Imputation will be performed separately by arm to avoid bias toward the null. Deaths will be recorded and imputed via MICE, with sensitivity analysis excluding deceased patients. For ICU transfers within 48 h or early discharges (before POD 3), MICE will be applied to available data, with subgroup or sensitivity analyses assessing robustness.

Multiplicity control: To control for multiplicity across secondary endpoints, the Bonferroni–Holm method will be applied within two pre-specified endpoint families, Family A: High-output stoma incidence, time to output stabilization, hypokalemia and Family B: Total potassium supplementation, index length of stay, total length of stay.

Definition transparency: The ITT, mITT, and per-protocol populations will be illustrated using decision-tree diagrams in the statistical analysis appendix to ensure reproducibility of analytical decisions.

Ethics and dissemination: All procedures in this study will be conducted in accordance with the ethical standards established by the institutional and national research committees, the General Data Protection Regulation, and the 1964 Declaration of Helsinki, along with its subsequent amendments or other comparable ethical guidelines. The study has been approved by the Bioethics Committee of the Medical University of Gdańsk (approval no. KB/99/2025). Ethics approval was obtained prior to the recruitment of participants. Informed consent, in writing, is required from all participants. The results of this study will be disseminated in a timely manner through academic conferences and peer-reviewed journals. The study protocol and clinical research data will be made publicly available following the publication of the primary manuscript in a peer-reviewed journal. These data will be provided on reasonable request, with further details to be included in the main manuscript.

Patient and Public Involvement: As this manuscript presents a protocol for a randomized controlled trial in patients with high-output stoma, patients or the public were not involved in the methodological design of the study. Nevertheless, the outcomes are expected to contribute to improving the quality of care and management of patients with high-output stomas. Study results will be disseminated not only in scientific publications and conferences but also through popular science forums and patient group networks to support wider awareness and enhance the quality of treatment.

## 4. Discussion

High-output stoma remains a frequent and clinically important complication after the creation of an ileostomy, causing fluid and electrolyte losses, secondary hyperaldosteronism, hypokalemia, hyponatremia and AKI, which in turn precipitate unplanned care and readmissions [7,32]. A recent review characterizes HOS as a “secretory” complication and reports incidences in the range of ~14–24% when defined by outputs between 1.0 and 2.0 L/day over several days, underscoring both the burden and variability introduced by non-uniform thresholds. Post-ileostomy readmission for dehydration or renal failure is common [33]; in historical series, ~17% of patients were readmitted within 30 days specifically for these indications, and AKI has been recognized as a frequent readmission diagnosis in contemporary datasets [7,8,33,34]. Major society guidance and practice updates emphasize structured fluids and electrolyte management, oral rehydration solutions with adequate sodium concentration, and early use of antimotility and antisecretory agents as core elements of care, reflecting convergence of expert practice even as high-grade trial evidence remains limited [10,21,35].

The physiologic rationale supports the use of proton pump inhibitors to mitigate effluent in settings of net intestinal secretion. In patients with extensive small-bowel resection, a population similar to early high-output ileostomies, intravenous omeprazole increased net water and sodium absorption versus no therapy or ranitidine in a randomized crossover experiment, with the greatest effect in those with high outputs (“net secretors”) [16]. Narrative and practice-oriented reviews for HOS management list PPIs alongside loperamide and codeine in stepped algorithms, aiming to reduce gastric and small-bowel secretions and thereby decrease effluent volume [10,35]. However, to our knowledge, no randomized trial has tested intensified PPI dosing in the immediate postoperative ileostomy population to prevent or attenuate HOS; therefore, high-quality interventional data specific to this question are lacking. STOP-HOS-1 is designed to address this gap.

The trial focuses on a clinically early postoperative window when output is most unstable and fluid or electrolyte disturbances carry the greatest clinical consequences. It compares an intensified intravenous omeprazole regimen with standard dosing in a pragmatic, multicenter, randomized, open-label design. The quantitative primary endpoint (mean 24 h output across POD 1–3, with a prespecified clinically meaningful difference of ≥250 mL/day) was selected because it reflects a physiological parameter directly informing bedside decisions, including oral fluid restriction, intravenous supplementation, and timing or escalation of antimotility therapy.

The targeted effect size, therefore, represents a pragmatically defined and clinically actionable reduction aligned with thresholds commonly guiding supportive management in routine practice.

Allocation concealment, standardized perioperative care pathways (including electrolyte monitoring, oral rehydration solutions, and stepwise antimotility therapy), a unified eCRF, and dedicated data collectors were implemented to reduce bias and improve measurement reliability, addressing methodological limitations frequently encountered in observational studies of ileostomy output. Nevertheless, the pragmatic perioperative setting requires individualized supportive care. Although fluid therapy, dietary adjustments, and antimotility escalation are prospectively recorded within a structured monitoring framework, variability in post-randomization clinical management cannot be entirely eliminated and should be considered when interpreting treatment effects. While the open-label design may introduce performance effects, the primary endpoint is objective and supported by prespecified intention-to-treat and per-protocol analyses with sensitivity assessments. At the same time, ileostomy output—measured through routine bedside assessment—may be subject to non-differential inter-observer variability, potentially attenuating detectable between-group differences. Moreover, the primary endpoint represents a physiological surrogate rather than a direct patient-centered outcome, and follow-up is limited to early postoperative recovery; longer-term quality of life and morbidity therefore remain beyond the scope of the present study. Inclusion of both elective and emergency procedures enhances external validity but introduces clinical heterogeneity reflecting real-world surgical populations. Because the study is not powered for definitive subgroup comparisons, any apparent variation in treatment effect across surgical contexts should be interpreted cautiously as exploratory. Similarly, incomplete POD 1–3 measurements related to early discharge, clinical deterioration, or perioperative mortality may occur despite prospective monitoring. Multiple imputation and prespecified sensitivity analyses were incorporated to mitigate bias related to missing data, although imputed values inherently introduce additional uncertainty, particularly when treatment effects are modest. The modified intention-to-treat framework further reflects pragmatic feasibility while requiring transparent interpretation with respect to potential attrition-related bias.

The study is adequately powered for a physiological endpoint, whereas secondary outcomes—including HOS incidence, dehydration-related complications, and 30-day readmissions—are analysed exploratorily; observed associations should therefore be considered hypothesis-generating pending confirmation in adequately powered trials. Conduct within two academic centers may also influence generalizability where postoperative pathways differ, despite standardized guideline-based management.

Given that both groups receive perioperative PPI prophylaxis as part of standard care and short-term intensified dosing has not been associated with a materially increased risk of serious adverse events, safety monitoring in STOP-HOS-1 prioritizes the detection of unexpected or imbalanced adverse events rather than predefined numerical stopping thresholds for rare complications [26].

Positive findings would be readily implementable and could strengthen the evidentiary basis for HOS management algorithms that currently rely largely on physiologic reasoning and lower-grade evidence. Conversely, neutral results would be equally informative, prompting the reconsideration of routine PPI escalation in early postoperative ileostomy care and redirecting attention toward alternative or combined strategies, including optimized oral rehydration, structured antimotility pathways, or phenotype-targeted treatment approaches such as biologically defined “net secretor” populations. In sum, STOP-HOS-1 directly tests an intuitive, low-cost, and scalable intervention embedded in routine postoperative care. By anchoring decision-making to quantitative effluent outcomes and clinically relevant complications, the trial aims to generate evidence capable of either reinforcing current practice where justified or recalibrating it where benefit is not supported by high-quality data.

Trial Status: The first version of the protocol was registered at ClinicalTrials.gov (NCT06917963) on 5 April 2025. Recruitment started on 10 April 2025 and is expected to conclude by July 2027.

## Figures and Tables

**Table 1:** Inclusion and exclusion criteria.

Category	Item	Description
Population		
	Target population	Adults undergoing the formation of a loop or end ileostomy (elective or emergency) at two academic centers in Poland (University Clinical Center, Gdańsk; PCK Maritime Hospital, Gdynia)
Inclusion Criteria		
	Age	≥18 years
	Surgery	Scheduled for formation of a loop or end ileostomy (elective or emergency)
	Consent	Able to provide written informed consent
	Drug suitability	No contraindication to omeprazole or other proton pump inhibitors
Exclusion Criteria		
	Pregnancy/lactation	Pregnancy or lactation
	Drug allergy	Known hypersensitivity or allergy to omeprazole or any other PPI
	Consent impossibility	Inability to provide written informed consent
	Competing trials	Concurrent participation in another interventional study likely to influence outcomes
	Output monitoring	Pre-existing conditions that preclude accurate 24 h stoma output measurement
	ICU/critical illness	Planned or immediate postoperative ICU admission with anticipated ICU stay > 48 h, or any critical illness in which early stomal output is frequently absent
	Investigator judgment	Any situation judged by the investigator to pose unacceptable risk or to confound assessment of the primary endpoint

**Table 2:** Intensified vs. standard omeprazole regimens.

Parameter	Intensified Omeprazole Arm	Standard Omeprazole Arm
Drug	Omeprazole	Omeprazole
Loading dose		
Drug	Omeprazole	Omeprazole
Dose	80 mg	None (standard care)
Route	Intravenous (IV)	N/A
Frequency	Single dose	N/A
Timing	Day before surgery	N/A
Maintenance dose (in-hospital)		
Drug	Omeprazole	Omeprazole
Dose	40 mg	40 mg
Route	Intravenous (IV)	Intravenous (IV)
Frequency	Twice daily (BID)	Once daily (OD)
Administration times	06:00 and 18:00 (±1 h)	06:00 (±1 h)
Duration	Through POD 10 or until discharge	During hospitalization only
Post-discharge continuation		
Drug	Omeprazole	None
Dose	40 mg	Not applicable
Route	Oral (PO)	Not applicable
Frequency	Twice daily (BID)	Not applicable
Duration	To complete 10-day course (if discharged before POD 10)	Omeprazole discontinued at discharge per ward standard protocol
Total treatment duration	10 days (Day of surgery through POD 10)	Variable (Day of surgery through discharge, typically 5–10 days)
Total daily dose		
Day before surgery	80 mg	0 mg
POD 1–10 (or until discharge)	80 mg/day	40 mg/day
Post-discharge (if before POD 10)	80 mg/day (oral)	0 mg/day (discontinued)
Cumulative dose (10-day course)	880 mg total	280–440 mg total (depending on length of stay)

**Table 3:** Patient flowchart—intensified omeprazole arm.

Study Period	Enrollment	Allocation	Post- Allocation		Allocation						Closeout
Timepoint	-t_1_	0	Day Before Surgery	POD 1	POD 2	POD 3	POD 4–7	POD 8–10	Discharge	POD 14	POD 30
Enrolment											
Eligibility screen	X										
Informed consent	X										
Medical history	X										
Physical examination	X										
Baseline labs (Na, K, Mg, Cr)	X										
Allocation		X									
Interventions											
Omeprazole 80 mg IV loading			X								
Omeprazole 40 mg IV twice a day				X	X	X	X	X			
Omeprazole 40 mg PO twice a day (if discharged before POD 10)							X	X	X		
Concomitant care											
24 h intake/output charting				X	X	X	X	X			
WHO oral rehydration solution				X	X	X	X	X			
Restriction of hypotonic fluids				X	X	X	X	X			
Electrolyte monitoring & supplementation				X	X	X	X	X			
Antimotility therapy (loperamide ± codeine)				X	X	X	X	X			
Dietetic education				X	X	X	X	X			
TPN if indicated				X	X	X	X	X			
Assesments											
Primary outcome											
24 h ileostomy output (mL/24 h)				X	X	X	X	X			
Mean output POD 1–3				X	X	X					
Secondary out-comes											
High-output stoma incidence				X	X	X	X	X			
Time to output stabilization				X	X	X	X	X			
Daily serum sodium				X	X	X	X	X			
Daily serum potassium				X	X	X	X	X			
Daily serum magnesium				X	X	X	X	X			
Serum creatinine (AKI assessment)				X	X	X	X	X			
Hyponatremia (Na < 135 mmol/L)				X	X	X	X	X			
Hypokalemia (K < 3.5 mmol/L)				X	X	X	X	X			
Acute kidney injury (KDIGO)				X	X	X	X	X			
Total potassium supplementation				X	X	X	X	X			
Antimotility therapy use				X	X	X	X	X			
TPN initiation and duration				X	X	X	X	X			
Length of stay (index admission)									X		
Patient-recorded output										X	X
Adverse events				X	X	X	X	X	X	X	X
30-day readmission											X
Total days hospitalized (30 days)											X

“X” indicates protocol-defined timing of enrolment procedures, interventions, concomitant care, or out-come assessments conducted or documented at the specified study timepoint.

**Table 4:** Patient flowchart—control arm.

Study Period	Enrollment	Allocation	Post- Allocation		Allocation						Closeout
Timepoint	-t_1_	0	Day Before Surgery	POD 1	POD 2	POD 3	POD 4–7	POD 8–10	Discharge	POD 14	POD 30
Enrolment											
Eligibility screen	X										
Informed consent	X										
Medical history	X										
Physical examination	X										
Baseline labs (Na, K, Mg, Cr)	X										
Allocation		X									
Interventions											
Omeprazole 80 mg IV loading				X	X	X	X	X			
Omeprazole 40 mg IV twice a day									X	X	
Omeprazole 40 mg PO twice a day (if discharged before POD 10)											
Concomitant care				X	X	X	X	X			
24 h intake/output charting				X	X	X	X	X			
WHO oral rehydration solution				X	X	X	X	X			
Restriction of hypotonic fluids				X	X	X	X	X			
Electrolyte monitoring & supplementation				X	X	X	X	X			
Antimotility therapy (loperamide ± codeine)				X	X	X	X	X			
Dietetic education				X	X	X	X	X			
TPN if indicated											
Assesments											
Primary out-come				X	X	X	X	X			
24 h ileostomy output (mL/24 h)				X	X	X					
Mean output POD 1–3											
Secondary outcomes				X	X	X	X	X			
High-output stoma incidence				X	X	X	X	X			
Time to output stabilization				X	X	X	X	X			
Daily serum sodium				X	X	X	X	X			
Daily serum potassium				X	X	X	X	X			
Daily serum magnesium				X	X	X	X	X			
Serum creatinine (AKI assessment)				X	X	X	X	X			
Hyponatremia (Na < 135 mmol/L)				X	X	X	X	X			
Hypokalemia (K < 3.5 mmol/L)				X	X	X	X	X			
Acute kidney injury (KDIGO)				X	X	X	X	X			
Total potassium supplementation				X	X	X	X	X			
Antimotility therapy use				X	X	X	X	X			
TPN initiation and duration									X		
Length of stay (index admission)										X	X
Patient-recorded output				X	X	X	X	X	X	X	X
Adverse events											X
30-day readmission											X

“X” indicates protocol-defined timing of enrolment procedures, interventions, concomitant care, or out-come assessments conducted or documented at the specified study timepoint.

## Data Availability

This article presents a clinical trial protocol; therefore, no research data were generated or analyzed for this publication. Upon completion of the study and publication of the primary results, de-identified individual participant data supporting the reported findings, together with the data dictionary and statistical analysis code, will be made available in accordance with institutional policies and GDPR requirements, either via a public repository or upon reasonable request to the corresponding author.
